# Evaluation and Comparison of Dermo-Cosmetic Activities of Three Oak Species by Targeting Antioxidant Metabolites and Skin Enzyme Inhibitors

**DOI:** 10.3390/metabo13070804

**Published:** 2023-06-28

**Authors:** Gaëlle Buche, Malorie Laffon, Laëtitia Fougère, Emilie Destandau

**Affiliations:** Institut de Chimie Organique et Analytique, Université d’Orléans-CNRS, UMR 7311 BP 6759, CEDEX 2, 45067 Orléans, France; gaelle.buche@univ-orleans.fr (G.B.); malorie.laffon@univ-orleans.fr (M.L.); laetitia.fougere@univ-orleans.fr (L.F.)

**Keywords:** mass spectrometry, molecular network, semi-preparative fractionation, in vitro antioxidant capability, in vitro enzymatic assays, sessile oak, pedunculate oak, pubescent oak

## Abstract

The two main species, sessile oak (*Quercus petraea* Liebl.) and pedunculate oak (*Quercus robur* L.), predominant in French forests, are mainly used for aging wines and spirits; however, the potential of oak wood extract as a source of natural antioxidants, due to its high polyphenol content, could be more widely exploited. This study focuses on three oak species, the two that are well-known, namely, sessile and pedunculate oak, and a third that has seldom been described and valorized, namely, pubescent oak (*Quercus pubescens*). Water extracts of these three species were fractionated by semi-preparative HPLC. The antioxidant activities of crude extracts and fractions were measured by colorimetric and enzymatic tests. The anti-elastase and anti-collagenase activities of the extracts and their fractions were also evaluated. In parallel, samples were analyzed by UHPLC-HRMS to correlate the activity with the molecular composition using molecular networks. The results obtained for the total extract of the three species were compared to determine if the activity depended on the species. The results within the same species were also compared to highlight which fraction and, therefore, which molecular family was involved in the activity of the total extract. The various antioxidant tests showed good activity of the total extract for the three species of oak and a very good anti-collagenase activity. The antioxidant activity of oak extract has already been proven in the literature and this is correlated with its richness in polyphenols. This study shows that each molecular family of the extract contributes to the activities of the total extract. Oak extract can be used to neutralize the ROS produced during oxidative stress and to prevent the degradation of collagen and elastase during skin aging. Its complementary properties make oak extract a valuable ingredient to act against skin aging.

## 1. Introduction

The genus Quercus includes no less than hundreds of oak species. Two species are predominant in French forests, namely, sessile oak (*Quercus petraea* Liebl.) and pedunculate oak (*Quercus robur* L.). Oak has always been used in several fields: oenology, food processing, and construction, while the ageing process in oak barrels is a common technological procedure used in winemaking, which appears to contribute to an increase in the antioxidant capacity of wines [1,2,3]. This indicates the potential of oak wood extract, which can be used as a source of natural antioxidants thanks to its richness in polyphenols; however, the molecular content of oaks is not limited to the polyphenolic fraction. Other molecular families such as lignanes, coumarins, and terpenoids are also present in oak extracts. This molecular diversity could be valorized in new ways to protect the skin against ageing. Cutaneous ageing is a complex biological phenomenon consisting of intrinsic factors, which are largely genetically determined, and extrinsic ones caused by environmental exposure to UV light, pollution, stress, etc. Skin aging is characterized by a degradation of collagen and elastin, leading to a decrease in the firmness and elasticity of the skin. In addition, oxidative stress causes the production of free reactive oxygen species (ROS), which accelerate skin ageing. Antioxidant molecules can be used to reduce and neutralize free radicals and can be combined with anti-collagenase or anti-elastase molecules to inhibit the activity of enzymes that degrade skin proteins [4,5]. The residues of wood transformation are a promising way to provide a high added value raw material source for natural antioxidant substances in order to develop cosmetic ingredients [6]. This study aims to investigate and compare the anti-aging potential of eco-friendly oak wood water extracts of three oak species for the first time. The two main oak species, namely, *Quercus petraea* Liebl. and *Quercus robur* L., were investigated and another oak species was also included, namely, *Quercus pubescens*, which is abundant in France, is known to resist drought more easily, but is not always of a good enough quality to be used in oenology [7,8]. The phytochemistry of this species is poorly described and no real valorization of its suitability as a cosmetic ingredient has yet been developed, apart from the activity of the leaves [9] and bark [10]. The antioxidant and anti-enzymatic activity of the three oak species in correlation with their phytochemical composition was, therefore, evaluated and compared. The antioxidant activity of crude extracts and fractions, obtained by semi-preparative HPLC, was evaluated on DPPH, ABTS, FRAP, CUPRAC and iron chelation in vitro assays. Moreover, to further investigate the anti-aging potential of the extracts and fractions, their capability to inhibit xanthine oxidase, elastase and collagenase was also studied for the first time. The extracts and fractions were analyzed by UHPLC-HRMS/MS to correlate their molecular composition with their activity.

## 2. Materials and Methods

### 2.1. Chemicals

The following reagents were purchased from Sigma-Aldrich (Saint Quentin Fallavier, France): 2,2-diphenyl-1-picrylhydrazyl (DPPH), potassium persulfate (99.99%), 3-(2-pyridyl)-5,6-diphenyl-1,2,4-triazine-p,p′-disulfonic acid monosodium salt hydrate (97%), copper (II) chloride (99.99%), 2,4,6-tris(2-pyriodyl)-s-triazine (TPTZ) (≥98%), sodium acetate (≥99%), 2,2′-azobis(2-methylpropionamidine) dihydrochloride granular (97%), trolox (≥98%), ferric chloride (97%), neocuproine (≥98%), glacial acetic acid, formic acid, ammonium acetate and ferric chloride hexahydrate. The 2,2′-azinobis-(3-ethylbenzthiazoline-6-sulfonic acid) (ABTS) (98%) reagent was purchased from ThermoFisher-Alfa Aesar, (Kandel, Germany).

The following enzymatic kits were purchased from Thermo Fischer Scientific (Waltham, MA, USA): EnzChek Gelatinase/Collagenase assay kit E-12055 and EnzChek Elastase assay kit E12056. Xanthine oxidase inhibition was measured using an assay kit 19160 purchased from Sigma Aldrich (Saint Quentin Fallavier, France).

The ultrapure water was produced with a PurelabFlex system from Veolia (Wissous, France). The acetonitrile used for the semi-preparative fractionation was HPLC grade, purchased from VWR, (Fontenay-sous-Bois, France). The acetonitrile from SDS Carlo Erba (Val de Reuil, France), used for the UHPLC-HRMS analysis, was a HPLC PLUS gradient grade.

The ethanol used for the oak wood extraction was HPLC grade and was purchased from VWR (Fontenay-sous-Bois, France). The formic acid used for the UHPLC-HRMS analysis was of an Optima LC-MS grade from Fisher Scientific (Illkirch-Graffenstaden, France).

### 2.2. Plant Material and Sample Preparation

Oak wood samples were provided by Tonnellerie Radoux and were a mix of different pieces of wood used for wine ageing. An amount of 10 g of oak wood samples dried and ground were extracted using ultrasound-assisted extraction with 150 mL of 100% water during 1 h. The extract was then filtered, frozen with liquid nitrogen and lyophilized. Samples from three heartwood species were used: pedunculate, sessile and pubescent oaks. The species of the oak wood samples were verified by genetic analysis [11]. The samples are extracted twice in order to check the repeatability of the extraction.

### 2.3. UHPLC-DAD-ELSD Analysis

Chromatographic analyses were performed using a Thermo Scientific Ultimate 3000 RSLC system equipped with an autosampler, a binary pump, a thermostated column compartment, a DAD detector (Dionex, Germering, Germany) and an ELSD Sedex 100LT detector (Sedere, Olivet, France) in dynamic gain. The mobile phase was composed of water (A) and acetonitrile (B) with both acidified with 0.1% of formic acid. Elution was performed in the gradient mode at a flow rate of 500 μL·min^−1^ and with the following binary gradient program: starting with 3% of solvent B during 0.2 min, 3–45%B from 0.2 to 12 min, 45–90%B from 12 to 14 min, and 90–3%B from 15 to 15.5 min. Then, the column was re-equilibrated with 3% of solvent B during 3 min. The column was a Luna^®^ Omega C18 (150 × 2.1 mm; 1.6 µm) (Phenomenex, Le Pecq, France,) heated at 40 °C and then 10 µL of the oak extracts and fractions were injected.

### 2.4. Fractionation by Semi-Preparative Liquid Chromatography

Semi-preparative experiments were performed with PCL 2250 from Gilson (Villiers le bel, France) with DAD detection. The column was a Nucleodur HTec C18 (250 × 25 mm; 5 µm) (Macherey-Nagel, Düren, Germany). The mobile phase was composed of water (A) and acetonitrile (B) with both acidified with 0.1% of formic acid. Elution was performed at a flow rate of 20 mL·min^−1^ and with the following gradient program: starting with 5% of solvent B, 5–20%B from 0 to 10 min, 20–30%B from 10 to 15 min, 30–40%B from 15 to 20 min, 40–50%B from 20 to 25 min, and 50–100%B from 25 to 30 min during 5 min. Then, the column was re-equilibrated with 5% of solvent B during 5 min. The oak extracts (100 mg) were solubilized in 4 mL of a water/ethanol mixture 85:15 *v*/*v* and injected for fractionation.

The collected tubes were pooled into 4 fractions according to their UHPLC-DAD-ELSD profile to separate the main molecular families in the different fractions and to recover enough raw material for each fraction to carry out bioactivity evaluations. The total extract and fractions were evaluated on 96-well plates by antioxidant and enzymatic assays. They were also analyzed using UHPLC-HRMS/MS.

### 2.5. In Vitro Antioxidant Capability

The antioxidant capability was evaluated using DPPH, ABTS, FRAP, CUPRAC and Iron (II) chelating assays. Generally, depending on the response of a sample in contact with reagents, a visual color change will be observed. This change will be reflected by a variation in the absorbance measurement. Here, a blank solvent, positive control, crude extract and the four fractions were tested for the 3 oak species.

The evaluation of antioxidant activity was performed in 96-well plates; each sample was deposited in triplicate. Three different sample volumes, i.e., 10, 5 and 1 µL of the 1 mg/mL extract and fraction solutions were deposited to give the final well concentrations of: 50 µg/mL, 25 µg/mL and 5 µg/mL. These concentrations were adapted to observe a dose/response effect and to avoid saturation of the absorbance measurement. Water was used to dilute the samples and to measure the blank solvent. Trolox was the positive control and was evaluated in cascading concentrations from 250 µg/mL to 0.5 µg/mL, with each deposited in triplicate. Each concentration of trolox was adapted per test to fit within the linearity range of the test response.

The absorbance was measured using a Clariostar microplate reader (BMG labtech, Champigny-sur-Marne, France).

The results are expressed as an inhibition percentage and calculated with reference to the blank.

#### 2.5.1. DPPH Radical Scavenging Activity Assay

DPPH assays were performed by modifying the method described by Lee et al. [12]. A 2 mM DPPH solution was prepared in ethanol and then diluted in ethanol to obtain a solution at 0.2 mM used for the assay. The sample volume (i.e., 10, 5 or 1 µL) was completed with water to obtain a final total sample volume of 10 µL. Then, the total volume of the samples (10 µL) and the DPPH reagent at 0.2 mM (190 µL) were mixed and incubated for 30 min in the dark at room temperature. The absorbance was recorded at 516 nm.

#### 2.5.2. ABTS Radical Cation Decolorization Assay

A modified ABTS protocol was used according to the method outlined by Tagliazucchi et al. [13]. The mixture of 7 mM of ABTS solution and 2.45 mM potassium persulfate solution in equal volumes was incubated in the dark for 16 h under agitation at room temperature. Next, 4 mL of the mixture was diluted with 50 mL of ethanol/water (25:75 *v*/*v*). The total volume of the samples or the blank (10 µL) was deposited and 190 µL of the diluted mixture was added. The absorbance was recorded after 30 min of incubation in the dark at room temperature at 734 nm.

#### 2.5.3. FRAP (Ferric Ion-Reducing Antioxidant Power) Assay

FRAP assays were carried out by readjusting the method described by Benzie and Strain [14]. Briefly, the FRAP reagent was composed of 300 mM acetate buffer in water, 20 mM of FeCl_3_ in a water/buffer mixture and 10 mM TPTZ solubilized in HCl 40 mM, mixed in a 10:1:1 (*v*/*v*/*v*) ratio. Thereafter, the total volume of the samples or the blank (10 µL) was mixed with 190 µL of the FRAP solution and incubated for 30 min in the dark at room temperature. The absorbance was measured at 590 nm.

#### 2.5.4. CUPRAC (Cupric Ion-Reducing Antioxidant Activity) Assay

A slightly modified CUPRAC protocol was used according to the method outlined by Apak et al. [15]. In short, the total volume of the samples or the blank (10 µL) was mixed with 190 µL of CUPRAC reagent prepared by combining 10 mM of a Cu(II) I buffer with 7.5 mM of neocuproine in ethanol and a 1 M acetate ammonium buffer in a 1:1:1 (*v*/*v*/*v*) ratio. The plate was incubated for 30 min in the dark at room temperature and the absorbance was measured at 450 nm.

#### 2.5.5. Iron (II) Chelating Assay

The ability of oak wood compounds to chelate Fe^2+^ was evaluated using a method described by Denis et al. with some modifications [16]. The reagent was prepared by combining 1 mM of FeCL_3_ and 0.3 mM of ferrozine in water at a 1:1 (*v*/*v*) ratio. The total volume of the samples or the blank (10 µL) was mixed with 190 µL of reagent and incubated for 30 min in the dark at room temperature. The absorbance was measured at 562 nm.

### 2.6. Enzymatic Tests

#### 2.6.1. Elastase Inhibition Assay

A 1X buffer was used to dilute the elastase at 0.5 U/mL. The enzyme substrate was prepared at 100 μg/mL and two concentrations of the inhibitor N-Methoxysuccinyl-Ala-Ala-Pro-Val-chloromethyl ketone were prepared at 0.01 mM and 0.04 mM.

On the plate, two controls were carried out: one positive control (with 100 µL of enzyme + 50 µL of 1X buffer), and one negative control (with 100 µL of enzyme + 50 µL of inhibitor) and a blank with water. A blank for each extract was also prepared by replacing the enzyme with the buffer. For the crude extracts and fractions, 2.5 μL of the samples at 10 mg/mL were mixed with 47.5 μL and 5 µL of the samples at the same concentration and were mixed with 45 μL of 1X buffer. Finally, 100 μL of elastase at 0.5 U/mL was added; therefore, two concentrations of samples were tested, namely, 125 µg/mL and 250 µg/mL (final well concentrations). The plate was incubated for 5 min at room temperature after the enzyme deposition. Finally, all the wells (controls and samples) were completed with 50 µL of substrate. The plate was incubated for 2 h at room temperature in the dark. The fluorescence (λ_excitation_ 477 nm − λ_emission_ 525 nm) was then measured. The results are expressed as an inhibition percentage and were calculated with reference to the blank.

#### 2.6.2. Collagenase Inhibition Assay

A 1X buffer was used to dilute the collagenase at 0.4 U/mL. The enzyme substrate was prepared at 125 μg/mL, and two concentrations of the reference inhibitor 10-Phenanthroline monohydrate were prepared at 0.2 mM and 0.5 mM.

On the plate, two controls were carried out: one positive control (with 100 µL of enzyme + 80 µL of 1X buffer), and one negative control (with 100 µL of enzyme + 80 µL of inhibitor) and a blank with water. A blank for each extract was also prepared by replacing the enzyme with a buffer. The first well concentration tested was 40 µg/mL. Due to response saturation, decreasing concentrations were then tested. For the extracts and fractions, 8 μL of the samples at 10 µg/mL and 1 µg/mL were mixed with 72 μL of 1X buffer and 100 μL of collagenase at 0.4 U/mL. Thus, 0.4 µg/mL and 0.04 µg/mL were the final well concentrations used to observe a dose response for this test, respectively. The plate was incubated for 5 min at room temperature after enzyme deposition. Finally, all the wells (i.e., the control and samples) were completed with 20 µL of substrate. The plate was incubated for 2 h at room temperature in the dark. The fluorescence (λ_excitation_ 483 nm − λ_emission_ 530 nm) was measured. The results are expressed as an inhibition percentage and were calculated with reference to the blank.

#### 2.6.3. Tyrosinase Inhibition Assay

The tyrosinase inhibition was determined according to the protocol of Lim et al. [17] Briefly, the buffer was prepared with 50 mL of water, 200 µL of NaOH 2 M and 290 µL of H_3_PO_4_ 1 M (pH 6.8). The enzyme was prepared at 125 U/mL, the L-Dopa substrate at 8.3 mM and the glabridin reference inhibitor at 500 µM. An amount of 10 µL of the crude extracts and fractions were mixed with 70 µL of the buffer. 

Then, 40 µL of tyrosinase enzyme (125 U/mL) were added to the samples, and 40 µL of buffer to the blank sample and the blank control. The enzyme was incubated for 5 min. Glabridin was used at 500 µM as the control inhibitor. Finally, 120 µL of the substrate (8.3 mM) were added to all wells. The plate was incubated for 30 min at room temperature in the dark. The absorbance was measured at 490 nm. The results are expressed as an inhibition percentage and were calculated with reference to the blank.

#### 2.6.4. Xanthine Oxidase Assay

A xanthine oxidase inhibition assay is an enzymatic assay based on the oxidation of xanthine to uric acid, producing superoxide radicals [18].

A positive control (i.e., 20 µL of enzyme + 20 µL of water) and a blank (i.e., 20 µL of buffer + 20 µL of water) were deposited on the plate. A blank for each extract was also prepared without the enzyme, which was replaced by the buffer.

For the extracts and fractions, 20 μL of the samples at 50 µg/mL and 100 µg/mL were mixed with 20 μL of xanthine oxidase. The final well concentrations tested were 4.2 µg/mL and 8.3 µg/mL to observe a dose response for this test, respectively. The plate was incubated for 5 min at room temperature after enzyme deposition. Finally, all the wells (i.e., the controls and samples) were completed with 200 µL of substrate. The plate was incubated for 20 min at room temperature in the dark and the absorbance was measured at 450 nm. The results are expressed as an inhibition percentage and were calculated with reference to the blank.

### 2.7. UHPLC-UV-MS/HRMS

Chromatographic analyses were performed using an Ultimate 3000 RSLC system equipped with an autosampler, a binary pump, a thermostated column compartment and a DAD detector (Dionex, Germering, Germany). The column was a Luna^®^ Omega C18 (150 × 2.1 mm; 1.6 µm) (Phenomenex, Le Pecq, France). The column temperature was set at 40 °C and 5 µL of the oak extracts and fractions were injected. The mobile phase was composed of water (A) and acetonitrile (B), both acidified with 0.1% of formic acid. Elution was performed at a flow rate of 500 μL·min^−1^ and with the following binary gradient program: starting with 3% of solvent B during 0.2 min, 3–45%B from 0.2 to 12 min, 45–90%B from 12 to 14 min, and 90–3%B from 15 to 15.5 min. Then, the column was re-equilibrated with 3% of solvent B during 3 min. The MS and MS/MS experiments were carried out on a maXis UHR-Q-TOF mass spectrometer (Bruker, Bremen, Germany) with an electrospray ion source (ESI), working in the negative ionization mode that allowed a better detection and isolation of the molecular ion compared to the positive ionization mode. The pressure of the nebulizing gas was set at 2 bar, and the flow rate and temperature of the dry gas were set at 9.0 L·min^−1^ and 200 °C, respectively. The capillary voltage was set at 4 kV. Mass spectra were summed during 400 ms in the *m*/*z* range 50–2250. All the MS data were processed using the DataAnalysis 4.4 software (Bruker). Molecular formulae were generated using the SmartFormula algorithm with an elemental composition of C, H, and O to an infinite number and N ≤ 4 with a mass accuracy ≤3 ppm, and they were submitted to the SciFinder, PubChem, Lotus and GNPS databases in order to propose compound structures.

MS/MS experiments were conducted using the Data Dependent Acquisition mode with 3 precursor ions in the *m*/*z* range 150–1600; ions were excluded after 6 s. The analysis included 2 segments: in the first one, from 0 to 8 min collision energies (CE) of 40 eV for monocharged ions and 30 eV for doubly charged ions were applied. In the second one, from 8 min to the end of the analysis, collision energies of 75 eV for monocharged ions and 40 eV for doubly charged ions were applied. The CE values were adapted to the kind of compounds: before 8 min, the molecules belonged to the tannin family, which were mostly composed of C-O bonds that are weaker than the C-C bonds of the terpenes, which eluted after 8 min. The CE values for doubly-charged ions are lower than for single-charged ones due to the proximity of both charges. The MS/MS spectra were summed during 400 ms; therefore, the total cycle time for the MS and MS/MS was 1.6 s.

### 2.8. Molecular Network Design

Molecular networks are visual representations of the chemical space present in tandem mass spectrometry (MS/MS) experiments by comparing the mass spectra in pairs to map an extract. Therefore, considering that ions with closed structures will give similar neutral losses or fragment ions, each cluster associates molecules with a similar fragmentation pathway that possess high structural similarities and are likely to belong to the same chemical family.

The bucket table was built using the Metaboscape 4.0 software (Bruker). The T-ReX 3D algorithm detected precursor ions with an intensity threshold of 10,000 a.u. and associated retention time, *m*/*z* and area for each analyte.

The mascot generic format (mgf) file and a quantification table were exported to the GNPS platform [http://gnps.ucsd.edu] access on 1 February 2022 using feature based molecular networking (FBMN) in order to build the molecular network [19,20]. The optimized parameters used for the molecular network design were: mass tolerance 0.02 Da for the parent and fragment mass, min pair cos 0.75, network TopK 10, maximum connected component size 100, minimum matched fragment ions 6, minimum cluster size 2, and yes run MSCluster.

The quantitative molecular networks were visualized using the Cytoscape 3.8.1 software [https://cytoscape.org] access on 1 February 2022. Within the network, one node corresponds to one MS/MS spectrum. Nodes are represented by pie charts. The proportion of each color in the nodes correlates with the relative abundance of the ion in each sample. The more abundant an ion is in a sample (i.e., the crude extract or fraction), the more dominant the color of this sample is in the pie chart. In the total molecular network, with the crude extract and the four fractions, the majority of ions within a single fraction are also highlighted. The size of the node is correlated to the intensity of the ion in the fraction. The majority of ions are represented with a larger node size.

### 2.9. Statistical Analysis

Statistical tests were performed to verify if the inhibition percentages were significantly different using an ANOVA on xlstat. Each fraction was compared to the crude extract per test and for each species with a Dunnett test. It was also checked if the inhibition percentages between the 3 species per test for the crude extract and each fraction were significantly different using pairwise Tukey tests. Activities were considered significantly different when *p* < 0.001. A correlation analysis and visualization of the data were performed using the Metaboanalyst 5.0 software. The hierarchical clustering heatmap was performed with mean-centered data to compare the level of extract and fraction activity. The Ward algorithm was used as the clustering method and hierarchical clustering was achieved using the Euclidean distance. Principal component analyses were performed using the FactoMineR package and Factoshiny library with R (v4.2.0). The data were centered and reduced to perform the PCA analysis.

## 3. Results and Discussion

The objective of this study was to assess the potential of oak heartwood extracts as anti-aging ingredients, to determine if the activity was dependent on the species of oak used, since the molecular composition can vary depending on the species, and to target the compound families involved in the activity. The literature reports that sessile oak is statistically richer in aromatic compounds while pedunculate oak is richer in tannins [21]. In addition, pedunculate oak contains compounds such as bartogenic acid or terpenes in dimeric forms while sessile oak contains other types of terpenes such as quercotriterpenosids that are glycosylated terpenes [22,23,24,25]. The molecular composition of pubescent oak remains undescribed.

To target anti-aging molecules, crude extracts of the three oak species were fractionated, the fractionation was then followed by UHPLC-DAD-HRMS/MS, and the molecular composition of each fraction was determined and compared to that of the crude extract using molecular networks. The different clusters highlighted the molecular families detected in each fraction.

The antioxidant and anti-enzymatic potentials of the total extract and the different fractions of the three species were then evaluated. The results obtained for the total extracts of the three species were compared to determine if the activity depended on the species. The results within the same species were also compared to highlight which fraction and, therefore, which molecular family was involved in the activity of the total extract.

### 3.1. Extract Fractionation and Fraction Analysis

**Figure 1:**
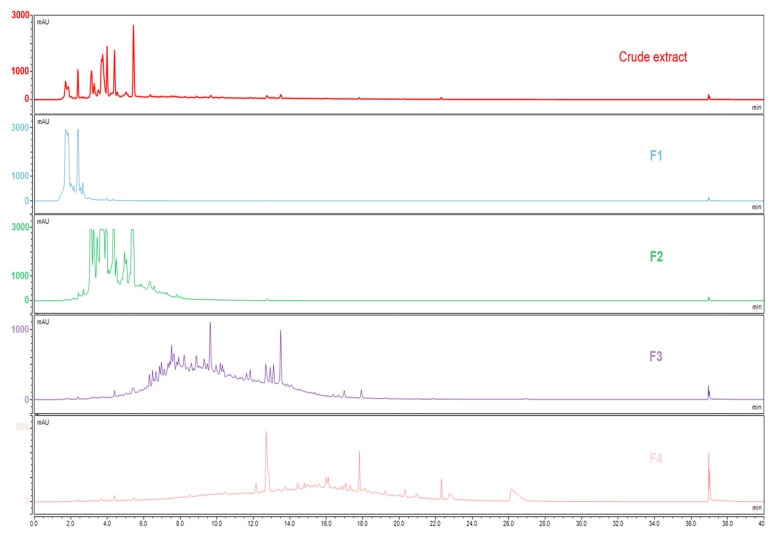
UHPLC-UV 280 nm analyses of the pedunculate crude extract (red), and the four fractions obtained by semi-preparative HPLC. Fraction 1 in blue, Fraction 2 in green, Fraction 3 in purple and Fraction 4 in pink. The Column Luna^®^ Omega C18 (150 × 2.1 mm; 1.6 µm), mobile phases water and acetonitrile were both acidified with 0.1% of formic acid in gradient elution mode, T 40 °C.

Each extract was fractionated by semi-preparative HPLC to separate the main molecular families according to their polarity related to their retention time on the C18 column. The four fractions obtained were dried and weighed and then prepared at the same concentration of 10 mg/mL in water. The HPLC-UV-ELSD chromatograms were acquired, and the results obtained at 280 nm for the crude extract and fractions of the pedunculate oak are shown in Figure 1. The same fractionation was obtained for the other two oak species.

The chromatogram of the crude extract in black shows major compounds eluted up to 6 min. The chromatograms of the different fractions point to a lower concentration of the less-polar compounds eluted after 6 min in Fractions 3 and 4 that were detected in very low amounts in the crude extract. Two complementary detectors were used: UV and ELSD. Both detections were weak for these apolar compounds, which means that they were present in small quantities in the crude extract. The compounds were generally well distributed between the fractions according to their polarity, although there was some overlap, particularly for Fraction 3 that shared some polar compounds with Fraction 2 and some less-polar ones with Fraction 4.

**Figure 2:**
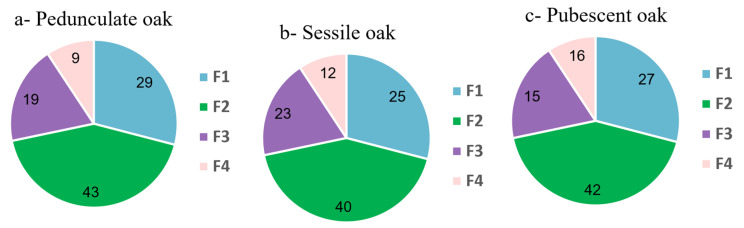
Percentages of weighed masses of the different fractions recovered after fractionation. Fraction 1 in blue, Fraction 2 in green, Fraction 3 in purple and Fraction 4 in pink.

The proportions of the dry masses obtained for the different fractions for the three species are presented in Figure 2.

In Figure 2a, the fractionation of pedunculate extract shows a highly-polar first fraction representing 29% of the total extract, with the compounds eluted at the void time and at very low retention times containing mainly primary metabolites (cf. Figure 1). The second fraction was the heaviest one; it represented 43% of the total extract due to the presence of tannins that absorbed well at 280 nm. The third fraction was lighter with 19% of the total mass and contained numerous compounds presenting a lower UV absorption at 280 nm or present in low concentrations. The last fraction represented 9% of the total extract and contained the most apolar compounds, some of which were also present in Fraction 3. Figure 2b,c presents quite similar fraction proportions for the sessile oak and pubescent oak, respectively. Fraction 1 of the sessile extract represented 25% of the total extract, while the second fraction was the heaviest once again, accounting for 40% of the total extract. The third fraction represented 23% of the total mass and the last fraction represented 12% of the total extract and contained the most apolar compounds. Fraction 1 of the pubescent extract represented 27% of the total extract, the second fraction 42%, the third fraction 15% and the last fraction 16%.

Overall, the mass distribution of the total extracts in the different fractions was the same for each of the three species. Fraction 2 was the heaviest, probably due to the presence of polyphenolic compounds, mainly tannins. Fraction 1 was the second heaviest fraction with the presence of highly-polar compounds such as sugars. The remaining two Fractions 3 and 4 were the lightest and contained the less-polar compounds, which presented a lower UV signal compared to the more-polar ones in Fraction 2 and they could have been terpenic compounds. For the sessile and pedunculate oaks, the lightest fraction was the most apolar Fraction 4, while for the pubescent oak, Fractions 3 and 4 were equivalent. In addition, the pubescent oak was less rich in Fraction 3 but richer in Fraction 4 compared to the other species.

### 3.2. Comparison of the Chemical Composition of Fractions and Crude Extract

**Figure 3:**
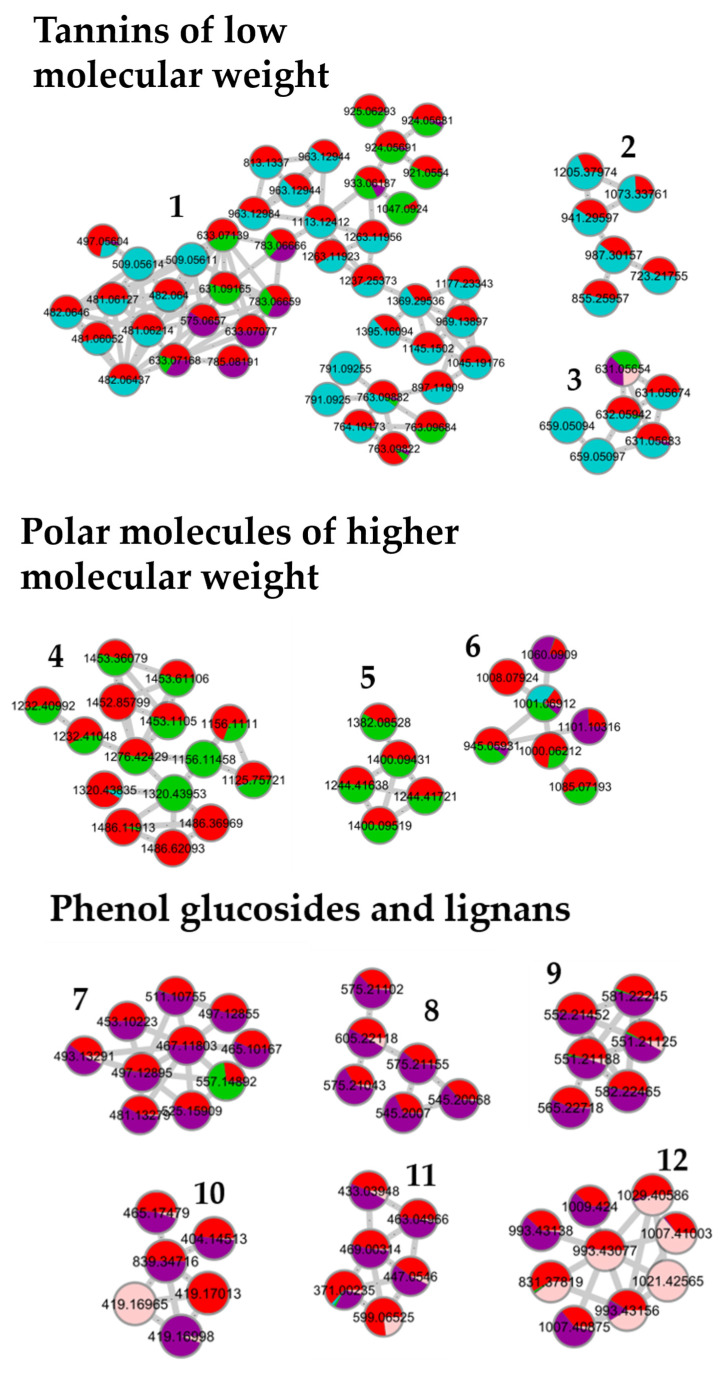

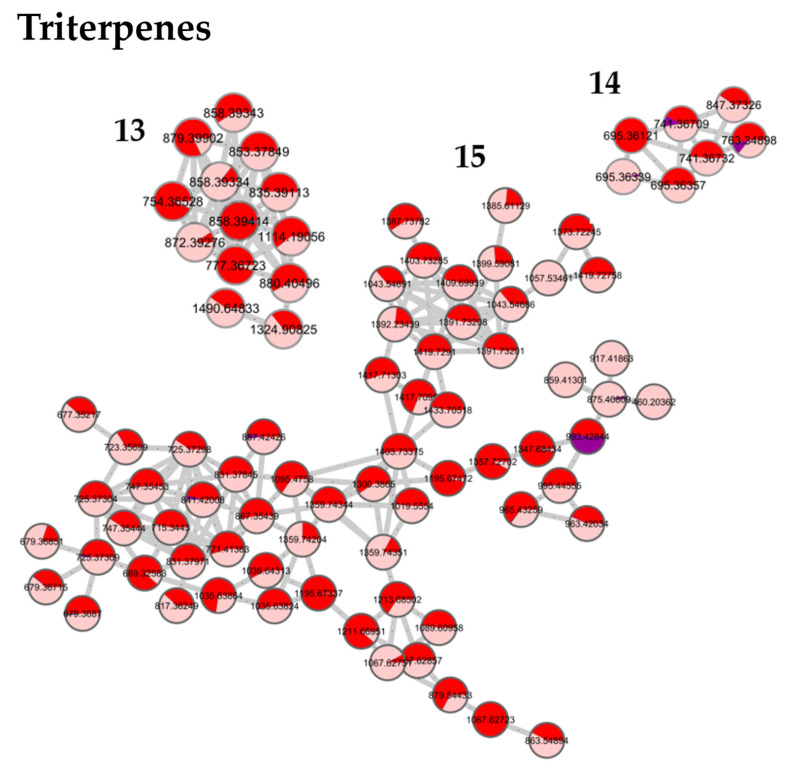
Mapping of pedunculate crude extract (red) and fractions (F1 in blue, F2 in green, F3 in purple and F4 in pink) with cos 0.75 and 6 matched fragment ions parameters.

The generation of the molecular network was based on the mass spectrometry analysis of the crude extracts and fractions. The MS/MS spectra of the compounds were compared pairwise to find similarities in their fragmentation pathways, i.e., the same fragment ions or similar neutral losses. To obtain suitable MS/MS spectra with a convenient number of fragment ions, the collision energies were optimized to find the ones that enabled fragmentation of the rigid structures while obtaining good spectra for the molecules that fragmented more easily. The molecules presenting similar fragmentation patterns were grouped in the same cluster [19,26,27]. As an example, Figure 3 shows the results obtained for the pedunculate oak. A similar distribution of fractions was obtained for the sessile and pubescent oak. According to the selected parameters, 13 clusters containing at least five nodes were formed. Based on previous work, on literature data, and on the interpretation of mass spectra and databases, the network was annotated [22,23,28]. Clusters 1 to 3 contained low molecular weight tannins and Clusters 4 to 6 polar molecules of a higher molecular weight. Clusters 7 to 12 were composed of phenols, glucosides, and lignans, while Clusters 13 to 15 contained triterpenes.

Clusters 1 to 12 were identical between the three species while clusters 13 to 15 were triterpenes derived from quercotriterpenosids for the sessile oak and derived from roburgenic acid for the pedunculate and pubescent oaks.

In Figure 3, the network of the crude extract (red) is associated with the network of each fraction to evaluate the distribution of molecules in the different fractions of each species. It confirms that only a small proportion of the molecules observed in the network were shared between several fractions. For example, ion *m*/*z* 783.0066 in Cluster 1 was distributed between Fraction 2 and 3 in a proportion that was rather similar; however, some molecules of a different polarity but belonging to the same cluster and, therefore, to the same molecular family, may have been eluted in different fractions. For example, Cluster 1 contained the most molecules eluted in Fraction 1 but also in Fractions 2 and 3.

Fraction 1 in blue, the most polar fraction, was divided into three Clusters 1, 2, and 3. It was composed of low-molecular mass ellagitannins with common fragments of ellagic acid such as the well-known vescalin/castalin suggested in Cluster 3 or other small-polar molecules such as HHDP glucose suggested in Cluster 1 [22].

Fraction 2 in green, was mainly composed of ellagitannins with a higher molecular weight than in Fraction 1, that were distributed in Clusters 1, 4, 5, and 6 with losses of vescalagin or ellagic acid [29].

Fraction 3 in purple, mainly grouped the Clusters 7, 8, 9, 10, and 11. These clusters highlighted molecular families of polyphenols that differed from the first two fractions, namely, phenol glucosides such as 3,4,5-trimethoxyphenyl-(6′-O-galloyl)-O-*β*-glucopyranoside in Cluster 6 or lignans such as lyoniresinol in Cluster 10 [30]. Cluster 9 was composed of molecules that had lyoniresinol as a fragment [31].

These three fractions were composed of phenolic compounds. In accordance with the literature, the high content of ellagitannins in oak wood was well identified in the heaviest Fractions 1 and 2. Other phenolic compounds were identified in lower concentrations in Fraction 3. 

Fraction 4 in pink, was mainly composed of terpenes, which are a well-known component of pedunculate oak. They were distributed in Clusters 12, 13, 14, and 15, such as triterpenes in dimeric form of the roburosid family or derivatives from bartogenic acid.

For the other two species the distribution was similar. The first three fractions were equivalent to those of the pedunculate oak. Fractions 1 and 2 were the heaviest for each of the three species and were composed of polyphenols, in particular ellagitannins. Fraction 3, which was heavier in the sessile and lighter in the pubescent oak, was also common to the three species. It was composed of lignan and phenol glucosides in the molecular network. Fraction 4, whose proportion varied between the species from 9% in the pedunculate to 16% for the pubescent and 12% for the sessile oaks, also differed in its molecular composition according to the species. For the pedunculate and pubescent oaks, the same type of molecule was found, namely, triterpenes such as bartogenic acid derivatives. For the sessile oak, other types of terpenes were found, mainly glycosylated terpenes including quercotriterpenosides [22,23,25].

In summary, this strategy allowed the rapid identification of molecular families contained in the crude extract by clustering compounds according to their structures without interpreting all the MS spectra. Moreover, it made it possible to highlight the molecular distribution of the compounds in each fraction. The three oak extracts had many molecules in common, with all the common polar parts composed of sugars and polyphenols. The three extracts were composed of low- and high-molecular mass ellagitannins, lignans and phenol glucosides. Finally, it was the most apolar fraction containing terpenic compounds that differed the most between the species; however, this fraction only represented about 10–15% of the crude extract and even if the molecules differed, they were all derived from pentacyclic triterpenes of the oleanolic acid type.

### 3.3. In Vitro Antioxidant Capability

A complete evaluation of the antioxidant activity of the oak crude extracts and all the semi-preparative fractions was performed using five different types of assays to measure the antioxidant properties. The assays were based on hydrogen atom transfer (DPPH and ABTS), single electron transfer mechanisms (FRAP, CUPRAC) or iron (II) chelating assays.

For each test, three concentrations of extracts were tested to verify the dose/response effect of the test. The whole results of the five antioxidant assays expressed as an activity percentage for a final sample concentration in the well of 25 µg/mL are presented in Table S1 in the supplementary data.

Crude extracts of the three species showed a good activity for each test. Some fractions also appeared to have an antioxidant activity.

For the crude extracts, the DPPH and ABTS tests showed a high percentage of inhibition for each of the three species. The results for the same test were similar, with about 72% of inhibition for the DPPH test and 96% for the ABTS test. While the reference standard trolox gave a 100% inhibition percentage at 40 µg/mL (i.e., the final concentration in well), the antioxidant activity for the ABTS test remained stronger than the DPPH test.

For the FRAP and CUPRAC tests, the activity results were more contrasted between the species with the highest activity for the pedunculate oak, then the pubescent and finally the sessile oak. For the FRAP test, there was very good activity between 80 and 90% while the activity of the CUPRAC test was less than this, ranging from 40 to 53%. Trolox gave similar inhibition percentages at 25 µg/mL for the FRAP and 50 µg/mL for the CUPRAC (i.e., the final concentration in well).

The activity for the iron chelation test was also very good and identical between the three species. The concentration in the reference standard trolox, which gave the same inhibition percentage, was around 50 µg/mL.

**Figure 4:**
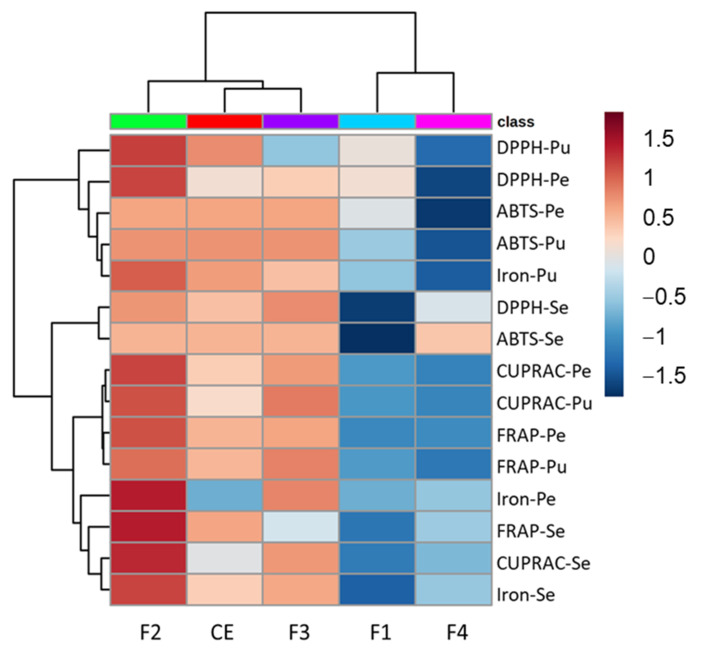
Heat map showing correlation between 5 antioxidants activities and samples at 25 µg/mL. Activities are expressed as inhibition percentages of the 5 antioxidant assays per species for the 4 fractions (F1 to F4) and the crude extract (CE). Dark red indicates a high inhibition percentage, while on the contrary, dark blue indicates a low inhibition percentage. Pu: Pubescent oak; Se: Sessile oak; Pe: Pedunculate oak.

Figure 4 presents the heat map correlating the crude extract, fractions and antioxidant activity.

The heat map shows a more similar behavior of the pubescent and pedunculate oaks that were clustered together while the sessile oak was dissociated from these two species for each activity test. The DPPH and ABTS assays based on the hydrogen atom transfer are clustered in the upper part of the heat map with the iron chelation for the pubescent oak, while the FRAP and CUPRAC based on single electron transfer mechanisms and iron chelation for the pedunculate and sessile oaks are clustered together in the lower part of the heat map, highlighting the similar mechanism involved for these tests. 

Fraction 2 showed the higher activity for most of the assays, in particular for the DPPH, CUPRAC and FRAP assays for the pedunculate and pubescent oak, as well as for the FRAP, CUPRAC and iron chelation for the sessile oak. Fraction 3 was also quite active with an intensity close to that of the crude extract. Fractions 1 and 4 were clustered together showing a lower activity. Fraction 1 showed a slight activity on the DPPH assay for the pubescent and pedunculate oaks, while Fraction 4 of the sessile was a little active on the ABTS assay. 

In summary, the various antioxidant tests showed good activity of the total extract for the three species of oak. The transfer of protons involved in the DPPH and ABTS tests was equally effective for all species, and the same holds for the iron chelation. On the contrary, the reducing potential was lower, especially for the CUPRAC test, which had the lowest response compared to the other four tests. Moreover, for each assay, the oak extracts presented a higher or similar antioxidant capability than the reference standard trolox that demonstrated the good potential of these extracts.

The antioxidant activity has already been proven in the literature to be correlated with polyphenol oak richness [32].

For the fractions, the activity was generally greater for Fractions 2 and 3, correlated with the molecular composition of these fractions, which were rich in tannins including vescalagin or castalagin derivatives for Fraction 2 and phenol glucosides for Fraction 3 [29].

In all the tests, the activity of Fraction 1 of the sessile oak was generally lower than that of the other species and Fraction 3 was also found to be less active in the FRAP test.

Fraction 4 of the pubescent oak appeared to be less active than for the other two species. The pedunculate and pubescent oaks showed, for the crude extract and fraction, a similar behavior for the evaluated assays, while the sessile oak presented, for some of them, different results. In addition, each fraction participated in the overall activity of the total extract, which involved several different molecular families in the antioxidant response [33].

### 3.4. Enzymatic Tests

To assess the anti-aging potential of the oak extract, the crude extracts and fractions were tested on three enzymes: xanthine oxidase, which is involved in the defense mechanism of ROS, and collagenase and elastase, which are involved in the loss of elasticity of the skin caused by the degradation of collagen and elastin.

Preliminary tests excluded tyrosinase which was not inhibited by the crude oak extract.

Table S2 in the supplementary data presents the results of the three enzymatic assays expressed as inhibition percentages. Several concentrations of extracts were tested to verify the dose/response effect of the tests. These concentrations were adapted to each test according to the response.

Good xanthine oxidase activity was measured with low tested concentrations. The three species were very active with an inhibition percentage of 99%, which saturated at 8.5 µg/mL. At this concentration all the fractions were highly active. The concentration had to be reduced to 4 µg/mL to observe a dose/response effect. Fraction 1 appeared to be less active compared to the crude extract for each species. These results complemented the previous antioxidant colorimetric tests showing good activity of the extracts.

The extracts as well as the fractions showed a weak elastase inhibition. The pedunculate and pubescent oaks were the most active species with 50% of inhibition at 250 µg/mL. At 125 µg/mL the activity was very low, presenting less than 15% of inhibition. In comparison, the reference inhibitor presented 73% of inhibition at 20 µg/mL. Fractions 2, 3 and 4 took part in the activity with an inhibition percentage between 25 and 30% while Fraction 1 was less active. The elastase inhibition of the sessile oak was half (20% of activity at 250 µg/mL) that of the other two species. It was less active on all fractions compared to the other two species.

The strongest activity was anti-collagenase inhibition which reached high percentages at very low tested concentrations of 0.4 and 0.04 µg/mL, while the reference inhibitor was around 80% of inhibition at a concentration of 47 µg/mL. The sessile oak appeared to be a little less active than the other two species. Fractions 2 and 3 were the most active. The least active fraction for the sessile oak was Fraction 4 while for the pedunculate and pubescent oak it was Fraction 1. Overall, each fraction participated in the activity of the crude extract even if Fractions 2 and 3 were more active [29].

The differences in activity highlighted with Fraction 4 may be linked to the molecular composition of this fraction, which differed for the sessile oak compared to the other two species; the pedunculate and pubescent oak were richer in triterpenes derived from roburgenic acid.

**Figure 5:**
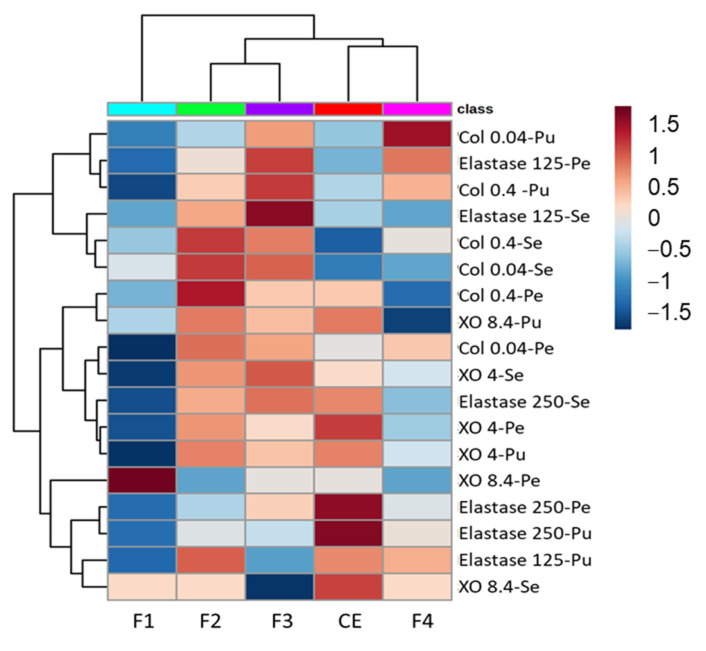
Heat map showing correlation between 3 enzymatic activities and samples. Activities are expressed as inhibition percentages of the 3 enzymatic assays per species for the 4 fractions (F1 to F4) and the crude extract (CE). Two concentrations expressed in µg/mL were tested for each 3 enzymatic assays. Dark red indicates a high inhibition percentage, while on the contrary, dark blue indicates a low inhibition percentage. Pu: Pubescent oak; Se: Sessile oak; Pe: Pedunculate oak; XO: Xanthine oxydase; Col: Collagenase.

Figure 5 presents the heat map correlating the inhibition potential of the crude extracts and fraction to the enzymatic assays.

Unlike antioxidant activity, no clustering according to the oak species or enzyme tested showed any real similarity or difference in behavior; nevertheless, Fraction 1 showed the lower participation to the inhibitory effect for the main experiments. Fractions 2 and 3 were the most involved fractions, while the Fraction 4 contribution was enzyme- and oak species-dependent.

In conclusion, the colorimetric tests, as well as the xanthine oxidase enzymatic test, showed an overall good antioxidant activity for the three species of oaks. There was only a low difference in response depending on the species, with an activity for the sessile oak being slightly lower, showing that the antioxidant activity was not drastically species-dependent. There were few differences between the fractions, showing that each molecular family participated in the activity of the total extract, even if the compounds contained in Fraction 2 appeared to be mainly involved due to their polyphenolic structure and their abundance.

Whatever the species, the oak extracts presented a rather low inhibition of elastase while the inhibition of collagenase was more efficient at a lower concentration. More differences between the species’ behavior were observed for the enzymatic assays. Sessile oak responded less well in those tests compared to the other two species. This may be due to its most-polar and most- non-polar fractions that showed lower activity than the other fractions. The terpene composition of Fraction 4 was different from that of the pubescent and pedunculate oaks. The pubescent and pedunculate oaks showed more similar behaviors, with a slightly higher activity of the pubescent oak at low concentrations.

### 3.5. Correlation between Molecular Content and Activity

**Figure 6:**
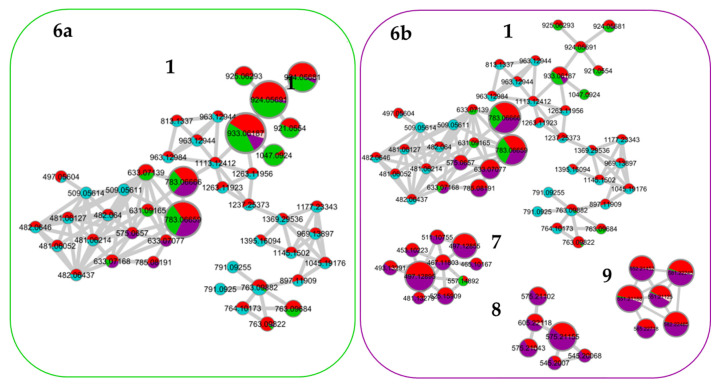
Quantitative molecular network of Fraction 2 (6a) in green and Fraction 3 (6b) in purple associated to the crude extract in red. In Figure 6a, the larger nodes represent the most abundant ions in Fraction 2 and in Figure 6b, the most abundant ions in Fraction 3.

Based on the molecular network of the crude extract with its four fractions presented in Figure 3, the most abundant molecules in Fractions 2 and 3 have been highlighted in Figure 6. Indeed, it was previously shown that the fractionation of the extract was undertaken by the molecular family. Within the same molecular family, compounds have similar structures and, thus, similar ionization yields can be assumed. The molecules with the strongest ion intensity in Fractions 2 and 3 are represented by a larger node size in Figure 6a,b, respectively.

Cluster 1 contained few major molecules contained in both Fraction 2 and 3 with a common molecule *m*/*z* 783.0665. Other abundant molecules were found for Fraction 2 in the single nodes. For Fraction 3, the majority of molecules were found in Clusters 7, 8, 9 and 10. Using the databases, the interpretation of the mass spectra and the literature, putative identifications were proposed for the most intense molecules in Fractions 2 and 3, and these are presented in the supplementary data Table S3. The most abundant molecules of Fraction 2 were ellagitannins such as Roburin derivatives, Grandidin, Castalagin, Vescalagin and Pedunculagin. Fraction 3 also presented high amounts of lignane such as Lyoniside and Lyoniresinol derivatives [29,31,34,35,36,37].

**Figure 7:**
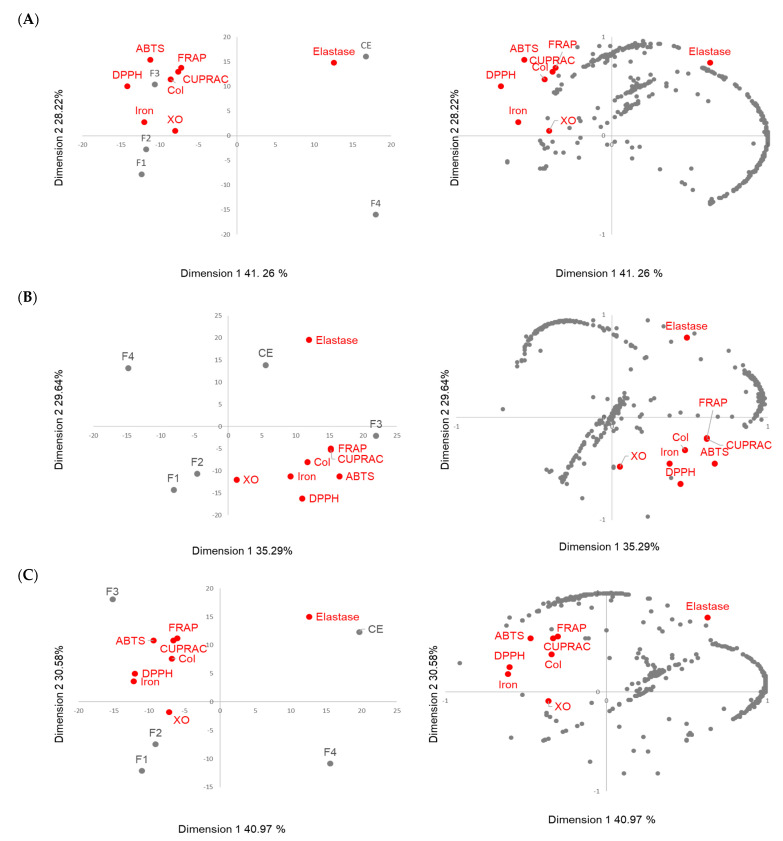
Principal component analysis (PCA) for the discrimination of the different fractions and crude extract of (**A**) pedunculate oak, (**B**) sessile oak, and (**C**) pubescent oak in the function of their phytochemical compositions and biological activities. The discrimination is represented by a biplot of the samples with the activities and loadings plot (with activities and intensities of the ions above the intensity threshold of 10^5^).

To correlate the antioxidant activity to the molecular composition of the oak extract intensity of the ions obtained using UHPLC-HRMS, the percentages of antioxidant and enzymatic activities were examined using a principal component analysis. Figure 7 presents the biplot and the loading plot obtained for the three species.

For each of the three species, the first two principal components (PCs) explained around 70% of the initial variance. The pedunculate and pubescent oaks had more similar behavior, and the representations were close (Figure 7A,C). The CE and F4 were projected in a PC1-positive score while F1, F2 and F3 were projected in a PC1-negative score that represented 41% of the variance for both species. The CE and F3 were projected in a PC2-positive score while F1, F2 and F4 were projected in a PC2-negative score, representing around 30% of the variance. The antioxidant and collagenase activities were mainly correlated with Fraction 3 and the XO activity was close to Fraction 2. Elastase was projected in the opposite side of the PC1 axe near to the crude extract. Fraction 1 was projected in the same side of the PC1 but was far from the activities on the PC2 axis, and Fraction 4 was projected completely away from the activities. Among the molecules projected near the antioxidant activities, the presence of lignanes such as Lyoniside belonging to Clusters 7 to 11 of the molecular network, and ellegitannin derivatives such as Pedunculagin found in Cluster 1, were observed and could contribute to the antioxidant activity of the oak extracts. Concerning the molecules projected close to the elastase, mainly compounds such as bartogenic acid derivatives belonging to the triterpene family found in Clusters 13 and 15 were observed. The pedunculate oak extract appeared to show a greater number of molecules contributing to elastase activity than the pubescent oak extract.

The sessile oak (Figure 7B) differed from the pedunculate and pubescent oaks since elastase, but also the antioxidant activities, were projected in a PC1-positive score; however, the correlation of activities with the fractions remained the same since F3 was projected near the antioxidant activity, while CE was projected near the elastase one. Fractions 1 and 2 were also projected in a PC2-negative score, where F2 was quite close to the XO and Fraction 4 remained at the opposite side on the PC1 and PC2. On the loading plot, a fewer number of molecules were projected near to activities. Ions were found as a single node in the molecular network and these compounds had not been previously identified in oak extracts. 

Gathering the results obtained with the heatmap and ACP, Fractions 2 and 3 were the most active and the molecules they contained appeared to be the main contributors to the extract’s antioxidant activity. Fraction 2 mainly contained ellagitannins, which are well-known for their antioxidant activity. Fraction 3 mainly contained lignans and phenol glucosides, which are also known for their antioxidant activity [29,38,39]. These compounds may also have contributed to the strong collagenase inhibition observed for the oak extracts. The triterpenes of the pedunculate and pubescent oaks that are bartogenic acid derivatives appear to contribute to the elastase activity, unlike the quercotriterpenosids terpene derivatives of the sessile oak.

The comparison of the activities of the three main oak species, namely, sessile, pedunculate and pubescent oak, was carried out for the first time. Oak water extract proves to be an intriguing green ingredient demonstrating an interesting valuation of pubescent oak, which is still seldom exploited. It has both a strong antioxidant activity due to its richness in phenolic compounds such as ellagitannins or lignans and an average anti-elastase activity of 50–60% inhibition at 250 µg/mL, combined with a very strong anti-collagenase activity of around 80% inhibition at 0.4 µg/mL and 50 % at 0.04 µg/mL, that was newly evaluated here. For these two enzymes, the sessile oak species was less effective while the pubescent oak was a little more active than the pedunculate oak. 

The oak extracts are rich in different molecular families such as small-polar ellagitannin derivatives, ellagitannins with heaviest molecular weight, that represented the main concentrated molecules in the crude extract, lignans, phenol glucosides and triterpene derivatives. Even if polyphenolic compounds appeared to contribute substantially to the overall activity, few differences in activity between the fractions were observed, indicating that each molecular family contributes to the overall activity. It is, therefore, more interesting to value the total extract to achieve an optimal ingredient effectiveness and to decrease production costs.

Thus, oak water extract could be used to neutralize the ROS produced during oxidative stress and to prevent the degradation of collagen and elastin during skin ageing. Its complementary properties could make oak extract a valued multi-functional ingredient to act against skin ageing. It would be interesting to delve further in the development of a cosmetic ingredient by confirming the in vitro tests with tests on cell cultures or skin models and investigating other types of activity.

## Data Availability

The data presented in this study are available on request from the corresponding author. Data is not publicly available due to privacy.

## Supplementary Materials

The following supporting information can be downloaded at: https://www.mdpi.com/article/10.3390/metabo13070804/s1, Table S1: Inhibition percentage of the 5 antioxidant assays per species for the 4 fractions and the total extract. The final concentration tested was 25 µg/mL. Activity percentages of fractions that are identical to those of the crude extract (rows) are noted * for significant values *p* < 0.001 according to Dunnett’s test. Activity percentages that are identical for the same fraction among the 3 species (columns) have the same superscript letter for significant values *p* < 0.001 according to Tukey’s test, Table S2: Inhibition percentage of the 3 enzymatic assays per species for the 4 fractions and the total extract tested at 2 concentrations. Inhibition percentages of fractions that are identical to those of the crude extract (rows) are noted * for significant values *p* < 0.001 according to Dunnett’s test. Inhibition percentages that are identical for the same fraction among the 3 species (columns) have the same superscript letter for significant values *p* < 0.001 according to Tukey’s test, Table S3: Description and proposition of annotation of the majority molecules in the most active fractions 2 and 3.

## References

[1] Larrauri J.A., Sánchez-Moreno C., Rupérez P., Saura-Calixto F. (1999). Free Radical Scavenging Capacity in the Aging of Selected Red Spanish Wines. J. Agric. Food Chem..

[2] Canas S., Casanova V., Pedro Belchior A. (2008). Antioxidant Activity and Phenolic Content of Portuguese Wine Aged Brandies. J. Food Compos. Anal..

[3] Alonso Á.M., Castro R., Rodríguez M.C., Guillén D.A., Barroso C.G. (2004). Study of the Antioxidant Power of Brandies and Vinegars Derived from Sherry Wines and Correlation with Their Content in Polyphenols. Food Res. Int..

[4] Jenkins G. (2002). Molecular Mechanisms of Skin Ageing. Mech. Ageing Dev..

[5] Kohl E., Steinbauer J., Landthaler M., Szeimies R.-M. (2011). Skin Ageing. J. Eur. Acad. Dermatol. Venereol..

[6] Dróżdż P., Pyrzynska K. (2018). Assessment of Polyphenol Content and Antioxidant Activity of Oak Bark Extracts. Eur. J. Wood Prod..

[7] Gallé A., Haldimann P., Feller U. (2007). Photosynthetic Performance and Water Relations in Young Pubescent Oak (*Quercus pubescens*) Trees during Drought Stress and Recovery. New Phytol..

[8] Haldimann P., Gallé A., Feller U. (2008). Impact of an Exceptionally Hot Dry Summer on Photosynthetic Traits in Oak (*Quercus pubescens*) Leaves. Tree Physiol..

[9] Plainfossé H., Burger P., Azoulay S., Landreau A., Verger-Dubois G., Fernandez X. (2018). Development of a Natural Anti-Age Ingredient Based on *Quercus pubescens* Willd. Leaves Extract—A Case Study. Cosmetics.

[10] Nisca A., Ștefănescu R., Mocan A., Babotă M., Nicolescu A., Mare A.D., Ciurea C.N., Man A., Tanase C. (2023). A Comparative Analysis of Polyphenol Content and Biological Potential of *Quercus petraea* Matt. and *Q. pubescens* Willd. Bark Extracts. Forests.

[11] Guichoux E., Lagache L., Wagner S., Chaumeil P., Léger P., Lepais O., Lepoittevin C., Malausa T., Revardel E., Salin F. (2011). Current Trends in Microsatellite Genotyping. Mol. Ecol. Resour..

[12] Lee S.K., Mbwambo Z.H., Chung H., Luyengi L., Gamez E.J., Mehta R.G., Kinghorn A.D., Pezzuto J.M. (1998). Evaluation of the Antioxidant Potential of Natural Products. Comb. Chem. High Throughput Screen..

[13] Tagliazucchi D., Verzelloni E., Bertolini D., Conte A. (2010). In Vitro Bio-Accessibility and Antioxidant Activity of Grape Polyphenols. Food Chem..

[14] Benzie I.F.F., Strain J.J. (1996). The Ferric Reducing Ability of Plasma (FRAP) as a Measure of “Antioxidant Power”: The FRAP Assay. Anal. Biochem..

[15] Apak R., Güçlü K., Özyürek M., Karademir S.E. (2004). Novel Total Antioxidant Capacity Index for Dietary Polyphenols and Vitamins C and E, Using Their Cupric Ion Reducing Capability in the Presence of Neocuproine:  CUPRAC Method. J. Agric. Food Chem..

[16] Dinis T.C.P., Madeira V.M.C., Almeida L.M. (1994). Action of Phenolic Derivatives (Acetaminophen, Salicylate, and 5-Aminosalicylate) as Inhibitors of Membrane Lipid Peroxidation and as Peroxyl Radical Scavengers. Arch. Biochem. Biophys..

[17] Evaluation of Antioxidant, Antibacterial and Anti-Tyrosinase Activities of Four Macaranga Species—Science Direct. https://www.sciencedirect.com/science/article/abs/pii/S0308814608011862.

[18] Kweon M.-H., Hwang H.-J., Sung H.-C. (2001). Identification and Antioxidant Activity of Novel Chlorogenic Acid Derivatives from Bamboo (*Phyllostachys edulis*). J. Agric. Food Chem..

[19] Wang M., Carver J.J., Phelan V.V., Sanchez L.M., Garg N., Peng Y., Nguyen D.D., Watrous J., Kapono C.A., Luzzatto-Knaan T. (2016). Sharing and Community Curation of Mass Spectrometry Data with GNPS. Nat. Biotechnol..

[20] Feature-Based Molecular Networking in the GNPS Analysis Environment|Nature Methods. https://www.nature.com/articles/s41592-020-0933-6.

[21] Prida A., Boulet J., Ducousso A., Nepveu G., Puech J. (2006). Effect of Species and Ecological Conditions on Ellagitannin Content in Oak Wood from an Even-Aged and Mixed Stand of *Quercus robur* L. and *Quercus petraea* Liebl. Ann. For. Sci..

[22] Buche G., Colas C., Fougère L., Destandau E. (2021). Oak Species *Quercus robur* L. and *Quercus petraea* Liebl. Identification Based on UHPLC-HRMS/MS Molecular Networks. Metabolites.

[23] Buche G., Colas C., Fougère L., Giordanengo T., Destandau E. (2020). Untargeted UHPLC-Q-TOF-HRMS Based Determination of Discrimating Compounds for Oak Species *Quercus robur* L. and *Quercus petraea* Liebl. Identification. Phytochem. Anal..

[24] Marchal A., Prida A., Dubourdieu D. (2016). New Approach for Differentiating Sessile and Pedunculate Oak: Development of a LC-HRMS Method To Quantitate Triterpenoids in Wood. J. Agric. Food Chem..

[25] Marchal A., Dubourdieu D. (2016). Method for Identifying the Oak Species of an Oak Wood Sample. U.S. Patent.

[26] Frank A.M., Bandeira N., Shen Z., Tanner S., Briggs S.P., Smith R.D., Pevzner P.A. (2007). Clustering Millions of Tandem Mass Spectra. J. Proteome Res..

[27] Watrous J., Roach P., Alexandrov T., Heath B.S., Yang J.Y., Kersten R.D., van der Voort M., Pogliano K., Gross H., Raaijmakers J.M. (2012). Mass spectral molecular networking of living microbial colonies. Proc. Natl. Acad. Sci. USA.

[28] Schymanski E.L., Jeon J., Gulde R., Fenner K., Ruff M., Singer H.P., Hollender J. (2014). Identifying Small Molecules via High Resolution Mass Spectrometry: Communicating Confidence. Environ. Sci. Technol..

[29] Fraga-Corral M., Otero P., Echave J., Garcia-Oliveira P., Carpena M., Jarboui A., Nuñez-Estevez B., Simal-Gandara J., Prieto M.A. (2021). By-Products of Agri-Food Industry as Tannin-Rich Sources: A Review of Tannins’ Biological Activities and Their Potential for Valorization. Foods.

[30] Centrifugal Partition Chromatography Applied to the Isolation of Oak Wood Aroma Precursors|Elsevier Enhanced Reader. https://reader.elsevier.com/reader/sd/pii/S0308814613005153?token=E993B2DAF29A3BBE1B6F2EB9836E50326FC688D5DA80E6036E1EB04F1BC6F141B831A98064361AB4E5DE019E76585EDB&originRegion=eu-west-1&originCreation=20210707140737.

[31] Winstel D., Marchal A. (2019). Lignans in Spirits: Chemical Diversity, Quantification, and Sensory Impact of (±)-Lyoniresinol. Molecules.

[32] Alañón M.E., Castro-Vázquez L., Díaz-Maroto M.C., Gordon M.H., Pérez-Coello M.S. (2011). A Study of the Antioxidant Capacity of Oak Wood Used in Wine Ageing and the Correlation with Polyphenol Composition. Food Chem..

[33] González Burgos E., Gómez-Serranillos M. (2012). Terpene Compounds in Nature: A Review of Their Potential Antioxidant Activity. Curr. Med. Chem..

[34] Vivas N., Glories Y., Bourgeois G., Vitry C. (1996). The Heartwood Ellagitannins of Different Oaks (*Quercus* Sp.) and Chestnut Species (Castanea Sativa Mill.). Quantity Analysis of Red Wines Aging in Barrels. J. Des Sci. Tech. Tonnelerie.

[35] Saucier C., Jourdes M., Glories Y., Quideau S. (2006). Extraction, Detection, and Quantification of Flavano-Ellagitannins and Ethylvescalagin in a Bordeaux Red Wine Aged in Oak Barrels. J. Agric. Food Chem..

[36] Cammann J., Denzel K., Schilling G., Gross G.G. (1989). Biosynthesis of Gallotannins: Beta-Glucogallin-Dependent Formation of 1,2,3,4,6-Pentagalloylglucose by Enzymatic Galloylation of 1,2,3,6-Tetragalloylglucose. Arch. Biochem. Biophys..

[37] Dad G., Corbani A., Manitto P., Speranza G., Lunazzi L. (2004). Lignan Glycosides from the Heartwood of European Oak Quercus Petraea. J. Nat. Prod..

[38] Othón-Díaz E.D., Fimbres-García J.O., Flores-Sauceda M., Silva-Espinoza B.A., López-Martínez L.X., Bernal-Mercado A.T., Ayala-Zavala J.F. (2023). Antioxidants in Oak (*Quercus* Sp.): Potential Application to Reduce Oxidative Rancidity in Foods. Antioxidants.

[39] Lobiuc A., Pavăl N.-E., Mangalagiu I.I., Gheorghiță R., Teliban G.C., Amăriucăi-Mantu D., Stoleru V. (2023). Future Antimicrobials: Natural and Functionalized Phenolics. Molecules.

