# Supplementary material for: Evaluation and Comparison of Dermo-Cosmetic Activities of Three Oak Species by Targeting Antioxidant Metabolites and Skin Enzyme Inhibitors
Source: Metabolites. 2023 Jun 28;13(7):804. doi: 10.3390/metabo13070804 (PMC10384454; doi:10.3390/metabo13070804)
Supplement: Supplementary file 1 [file metabolites-13-00804-s001.zip › metabolites-2430503-supplementary.pdf]

Supplementary data

Table S1: Inhibition percentage of the 5 antioxidant assays per species for the 4 fractions and the total extract. The final concentration tested was 25 µg/mL. Activity percentages of fractions that are identical to those of the crude extract (rows) are noted * for significant values p<0.001 according to Dunnett's test. Activity percentages that are identical for the same fraction among the 3 species (columns) have the same superscript letter for significant values p<0.001 according to Tukey's test.

Colorimetric tests	Species	Crude extract	F1	F2	F3	F4
		% activity		% activity		
DPPH	Pedunculate	74^{*a}	74^{*a}	79^{a}	75^{*a}	66^{a}
	Sessile	70^{*b}	34^{b}	75^{b}	76^{a}	61^{b}
	Pubescent	72^{*ab}	62^{c}	78^{a}	76^{a}	43^{c}
ABTS	Pedunculate	96^{*a}	94^{*a}	96^{*a}	96^{*a}	86^{a}
	Sessile	96^{*a}	59^{b}	96^{*a}	96^{*a}	94^{*b}
	Pubescent	96^{*a}	72^{c}	96^{*a}	96^{*a}	53^{c}
CUPRAC	Pedunculate	53^{*a}	28^{a}	70^{a}	60^{a}	24^{a}
	Sessile	40^{*b}	13^{b}	72^{a}	57^{b}	25^{a}
	Pubescent	42^{*b}	14^{b}	65^{b}	59^{ab}	10^{b}
FRAP	Pedunculate	90^{*a}	58^{a}	106^{a}	93^{a}	49^{a}
	Sessile	80^{*b}	27^{b}	103^{b}	58^{b}	48^{a}
	Pubescent	88^{*a}	35^{c}	104^{ab}	100^{c}	24^{b}
Iron chelation	Pedunculate	97^{*a}	97^{*a}	108^{a}	105^{a}	98^{*a}
	Sessile	98^{*a}	86^{b}	104^{b}	100^{*b}	92^{b}
	Pubescent	103^{*b}	93^{c}	106^{ab}	101^{*b}	86^{c}

Table S2: Inhibition percentage of the 3 enzymatic assays per species for the 4 fractions and the total extract tested at 2 concentrations. Inhibition percentages of fractions that are identical to those of the crude extract (rows) are noted * for significant values p<0.001 according to Dunnett's test. Inhibition percentages that are identical for the same fraction among the 3 species (columns) have the same superscript letter for significant values p<0.001 according to Tukey's test.

Enzymatic tests	Concentrations tested	Species	Crude extract	F1	F2	F3	F4
			% Inhibition	% Inhibition			
Xanthine oxidase	8.4 µg/mL	Pedunculate	98*[a]	100*[a]	97*[a]	98*[a]	97*[a]
		Sessile	99*[a]	98*[ab]	98*[a]	96[a]	98*[a]
		Pubescent	99*[a]	96[cb]	99*[a]	98*[a]	93[b]
	4 µg/mL	Pedunculate	63*[a]	47[a]	60[a]	57[a]	53[a]
		Sessile	54*[b]	43[b]	57[b]	59[a]	52*[a]
		Pubescent	55*[b]	43[b]	55[*b]	53*[b]	47[b]
Elastase	250 µg/mL	Pedunculate	52*[a]	14[a]	26[a]	35[a]	30[a]
		Sessile	20*[b]	0[b]	18*[b]	21*[b]	8[b]
		Pubescent	56*[c]	8[c]	28[a]	25[c]	30[a]
	125 µg/mL	Pedunculate	4*[a]	0[a]	9[a]	16[a]	14[a]
		Sessile	2*[a]	0*[a]	7[b]	12[b]	0*[b]
		Pubescent	15*[b]	6[b]	16*[c]	8[c]	14*[a]
Collagenase	0.4 µg/mL	Pedunculate	79*[a]	70[a]	88[a]	79*[a]	65[a]
		Sessile	37*[b]	56[b]	94[b]	86[b]	68[b]
		Pubescent	74*[c]	62[c]	81[c]	90[c]	83[c]
	0.04 µg/mL	Pedunculate	49*[a]	21[a]	64[a]	59[a]	55[a]
		Sessile	21*[b]	40[b]	64[a]	60[a]	27[b]
		Pubescent	56*[c]	50[c]	58*[b]	69[c]	78[c]

Table S3: Description and proposition of annotation of the majority molecules in the most active fractions 2 and 3.

Fraction number	Cluster number	R_T (min)	Measured m/z $[M-H]^-$ or $[M-2H]^{2-}$	Formula [M]	Error (ppm)	MS/MS fragments $[M-H]^-$	Proposed annotation
Fraction 2	Single node	2.41	990.0807^{2-}	$C_{87}H_{58}O_{55}$	-2.4	1065.1068 $C_{46}H_{33}O_{30}$<br>915.0544 $C_{41}H_{23}O_{25}$	Roburin B or C [29,34]
	Cluster 1	2.95	924.0613^{2-}	$C_{82}H_{50}O_{51}$	-1.8	631.0587 $C_{27}H_{19}O_{18}$<br>300.9993 $C_{14}H_5O_8$	Roburin A or D [29,34]
	Single node	3.01	1065.1095^{1-}	$C_{46}H_{34}O_{30}$	-2.1	1021.1164 $C_{45}H_{33}O_{28}$<br>975.0763 $C_{43}H_{27}O_{27}$	Grandinin or roburin E [29,34]
	Single node	3.32	1100.0728^{1-}	$C_{48}H_{30}O_{31}$	-1.1	1057.0800 $C_{47}H_{29}O_{29}$<br>933.0635 $C_{41}H_{25}O_{26}$<br>631.0573 $C_{27}H_{19}O_{18}$	Vescavaloninic acid [35]
	Cluster 1	3.91	933.0645^{1-}	$C_{41}H_{26}O_{26}$	-0.8	631.0580 $C_{27}H_{19}O_{18}$<br>300.9991 $C_{14}H_5O_8$	Castalagin or vescalagin [29]
Fractions 2 and 3	Cluster 1	4.45	783.0693^{1-}	$C_{34}H_{24}O_{22}$	0.2	481.0633 $C_{20}H_{17}O_{14}$<br>300.9986 $C_{14}H_5O_8$<br>275.0186 $C_{13}H_7O_7$	Pedunculagin [36]
Fraction 3	Cluster 7	5.10	497.1304^{1-}	$C_{22}H_{26}O_{13}$	-0.6	313.0568 $C_{13}H_{13}O_9$<br>169.0140 $C_7H_5O_5$<br>125.0244 $C_6H_5O_3$	3,4,5-trimethoxyphenyl-(6′-O-galloyl)-O-β-glucopyranoside [30]
	Cluster 9	6.85	581.2256^{1-}	$C_{28}H_{38}O_{13}$	-0.3	419.1722 $C_{22}H_{27}O_8$	Lyoniresinol glucoside [37]
	Cluster 9	7.38	551.2159^{1-}	$C_{27}H_{36}O_{12}$	-0.3	419.1711 $C_{22}H_{27}O_8$	Lyoniside [37]
	Cluster 10	7.65	839.3509^{1-}	$C_{44}H_{56}O_{16}$	-2.0	419.1719 $C_{22}H_{27}O_8$	Lyoniresinol [31]
	Cluster 8	7.79	575.2136^{1-}	$C_{29}H_{36}O_{12}$	-0.4	331.1117 $C_{18}H_{19}O_6$<br>165.0557 $C_9H_9O_3$	Not identified
